# Genome-Wide Analysis of TCP Family Genes in *Zea mays* L. Identified a Role for *ZmTCP42* in Drought Tolerance

**DOI:** 10.3390/ijms20112762

**Published:** 2019-06-05

**Authors:** Shuangcheng Ding, Zhenzhen Cai, Hewei Du, Hongwei Wang

**Affiliations:** 1 Agricultural College, Yangtze University, Jingzhou 434025, China; shchding@yangtzeu.edu.cn (S.D.); 18339069671@163.com (Z.C.); duhewei666@163.com (H.D.); 2 Hubei Collaborative Innovation Center for Grain Industry, Yangtze University, Jingzhou 434025, China; 3 College of Life Science, Yangtze University, Jingzhou 434025, China

**Keywords:** maize, *ZmTCP*, natural variation, drought tolerance

## Abstract

The Teosinte-branched 1/Cycloidea/Proliferating (TCP) plant-specific transcription factors (TFs) have been demonstrated to play a fundamental role in plant development and organ patterning. However, it remains unknown whether or not the *TCP* gene family plays a role in conferring a tolerance to drought stress in maize, which is a major constraint to maize production. In this study, we identified 46 *ZmTCP* genes in the maize genome and systematically analyzed their phylogenetic relationships and synteny with rice, sorghum, and *Arabidopsis* *TCP* genes. Expression analysis of the 46 *ZmTCP* genes in different tissues and under drought conditions, suggests their involvement in maize response to drought stress. Importantly, genetic variations in *ZmTCP32* and *ZmTCP42* are significantly associated with drought tolerance at the seedling stage. RT-qPCR results suggest that *ZmTCP32* and *ZmTCP42* RNA levels are both induced by ABA, drought, and polyethylene glycol treatments. Based on the significant association between the genetic variation of *ZmTCP42* and drought tolerance, and the inducible expression of *ZmTCP42* by drought stress, we selected *ZmTCP42*, to investigate its function in drought response. We found that overexpression of *ZmTCP42* in Arabidopsis led to a hypersensitivity to ABA in seed germination and enhanced drought tolerance, validating its function in drought tolerance. These results suggested that ZmTCP42 functions as an important TCP TF in maize, which plays a positive role in drought tolerance.

## 1. Introduction

Severe drought causes a grievous decline in crop yield by negatively affecting plant growth and reproduction [1,2]. Maize (*Zea mays* L.) is considered a major crop for food, feed, and fuel, but its production is frequently hampered by water scarcity [3,4]. While the molecular mechanisms of drought stress response in plants have not been fully elucidated, many transcription factors (TFs) such as DREB1/CBF, MYB, and AREB/ABF, which regulate drought-responsive genes have been well-studied [5,6,7]. These reports support the idea that identification of key TFs will help us to better understand the molecular and cellular responses to drought stress.

*Teosinte branched 1/Cycloidea/Proliferating (TCP)* genes encode plant-specific TFs and are named after the first three functionally characterized members of this TF family—*Teosinte Branched 1* (*TB1*) in maize (*Zea mays* L.), *Cycloidea* (*CYC*) in snapdragon (*Antirrhinum majus*), and *Proliferating Cell nuclear antigen Factor* (*PCF*) in rice (*Oryza sativa*) [8]. This class of TFs share a highly conserved TCP domain, which contains a 59-amino acid, non-canonical basic-Helix-Loop-Helix (bHLH) structural motif that allows DNA binding, protein–protein interaction, and protein nuclear localization [8,9,10]. More than 20 TCP TF members were identified in various plant species, such as *Arabidopsis*, rice, tomato, cotton, sorghum, and wheat [11,12,13,14,15,16,17]. Based on the TCP domain, these genes were divided into two classes. Class I is the PCF class; class II is subdivided into two clades—CIN (*CINCINNATA* of *Antirrhinum*) and CYC/TB1 [10,15,18]. Many members of the TCP family have been shown to be involved in the regulation of many biological processes during plant growth and development, such as leaf development, branching, floral organ morphogenesis, and senescence [8,19,20,21,22]. Possible mechanisms for the regulation were studied. These mechanisms were found to involve either a direct transcriptional control of the cell cycle genes by TCP TFs, or an indirect adjustment of the hormone activity [23]. For example, in *Arabidopsis* seeds, the DELLA proteins GAI (GA-Insensitive) and RGA (Repressor of GA) formed an unproductive complex with the class II TCP protein AtTCP14 or AtTCP15, which prevented the binding of TCPs to promoters of the core cell cycle genes [24]. In turn, GA might induce ubiquitination and degradation of DELLA proteins to relieve the constitutive inhibition of TCP transcriptional activity [24]. Interestingly, the orthologous TCP protein LANCEOLATE was found to participate in GA biosynthesis by upregulating the *SIGA-oxidase 1* gene [25]. It was also found that a subset of class I TCP proteins, such as AtTCP1, AtTCP2, AtTCP3, and AtTCP14, behaved in a similar fashion by acting as an inducer or repressor to influence the biosynthesis or signaling of several hormones during different developmental processes [26,27,28].

Abscisic acid (ABA) is a known stress response hormone that can mitigate physiological and environmental stresses, including drought stress, by inducing the closure of stomata, thus reducing water loss [29,30,31,32]. Although the *TCP* gene family is primarily involved in the regulation of plant growth and leaf development, other functions, such as involvement in the ABA signaling pathway, have been noted for specific *TCP* genes. For example, overexpression of *OsTCP19* in *Arabidopsis* significantly conferred both drought and heat tolerance during seedling establishment and in mature plants [33]. Furthermore, the interaction of OsTCP19 with OsABI4, which encodes a TF involved in the ABA signal transduction, suggests its function in fine-tuning drought-induced ABA signaling [33]. Additionally, creeping bentgrass plants (*Agrostis stolonifera*) that overexpresses *Osa-miR319*, in which four putative target genes, *AsPCF5*, *AsPCF6*, *AsPCF8*, and *AsTCP14* are down-regulated, significantly enhance plant tolerance to salt and drought stress associated with an increased leaf wax content and water retention [34]. It is noteworthy that AtTCP14 antagonizes ABA signaling by interacting with the DOF6 (DNA binding with one finger) TF, preventing the activation of the downstream ABA biosynthetic gene *ABA1* (*ABA deficient 1*) and other ABA-responsive genes in *Arabidopsis* seeds [35]. In contrast, *AtTCP18*, also known as *BRC1* (*Branched 1*), induces *ABF3* (*ABA responsive elements-binding factor 3*), and *ABI5* (*ABA insensitive 5*)—two key regulators of the ABA response—to maintain ABA signaling when the axillary buds enter dormancy [36,37]. These reports indicate a strong association of TCPs with ABA-mediated abiotic stress signaling. However, how the TCP TF genes function in maize, especially in response to drought stress, still remains to be elucidated. Due to the rapid linkage disequilibrium (LD) decay in the maize genome, association study is able to provide a gene-level resolution, which facilitates the genetic detection of several complex traits, such as drought tolerance [38,39,40]. However, limited allelic variations underlying drought tolerance have been identified [41], and rarely favorable alleles could be used for the genetic improvement of drought tolerance in maize.

Here, we comprehensively analyzed the *ZmTCP* gene family in the maize genome. Previous research has identified 29 *TCP* genes in the maize genome and subsequently, phylogeny, gene structure, chromosomal location, gene duplication, and expression levels of the 29 *ZmTCP* genes were investigated [42]. In this study, we searched against the updated maize genome B73_RefGen_v3 and identified 46 *ZmTCP* genes, and further systematically analyzed to determine their phylogenetic relationships and synteny with rice, sorghum, and *Arabidopsis TCPs*. In addition, we studied the expression profiles of these *ZmTCP*s upon drought stress. Importantly, a family-based, genome-wide association study revealed a significant association between the natural variations of *ZmTCP42* and maize drought tolerance. Ectopic expression of *ZmTCP42* in *Arabidopsis* led to enhanced drought tolerance, validating its function in drought tolerance.

## 2. Results

### 2.1. The Maize Genome Contains 46 TCP Family Genes

We comprehensively performed a genome-wide search for putative *TCP* genes in maize. Initially, the protein sequences of the putative maize TCP TFs were retrieved from the Plant Transcription Factor Database 3.0 (available online: http://planttfdb.cbi.pku.edu.cn). Subsequently, a BLASTP analysis was performed in Phytozome V10 (available online: http://www.phytozome.net/eucalyptus.php), using all of the *Arabidopsis* and rice TCP protein sequences as queries, and the predicted protein sequences without a TCP domain (Pfam: PF03634) were excluded from the results. A Hidden Markov Model (HMM) search was also performed against the maize database using PF03634. Ultimately, 46 genes, including the 29 *ZmTCPs* previously reported by Chai et al. [42], were retrieved and further verified for the presence of a canonical TCP domain. The 17 newly identified *ZmTCPs* were named *ZmTCP30* to *ZmTCP46*. Their locus IDs, genome locations, coding sequence (CDS) lengths, and protein lengths are listed in Table 1. The 46 *ZmTCP* genes were unevenly distributed throughout the maize genome on 10 chromosomes, without any clustering. The 46 ZmTCP proteins contained a range of 98 to 778 amino acids, corresponding to 11.76 kDa to 84.78 kDa in molecular weight (Table 1). Their theoretical pI (isoelectric point) values ranged from 5.13 to 12.23, with a mean of 8.14 (Table 1), indicating that most of them were weakly alkaline (Table 1).

### 2.2. Maize Contains Roughly Twice as Many TCP Genes as Rice and Sorghum

**Figure 1:**
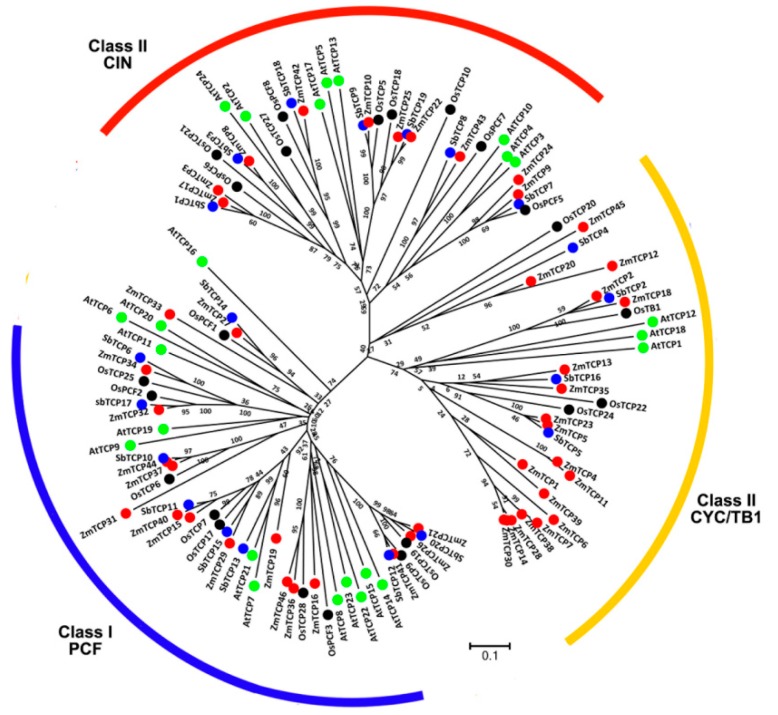
Phylogenetic tree of the predicted Teosinte-branched 1/Cycloidea/Proliferating (TCP) proteins from maize, rice, sorghum, and *Arabidopsis*. The phylogenetic tree was constructed based on the sequence alignment of the 113 full-length TCP protein sequences from four species. The unrooted tree was drawn by MEGA 7.0 with the neighbor-joining (NJ) method, using the following parameters—bootstrap values (1000 replicates) and the poisson model. The scale refers to the branch lengths. The gene codes and names are illustrated in red for maize, black for rice, blue for sorghum, and green for *Arabidopsis*. The names used for rice and the Arabidopsis *TCP* genes are from a previous report [8].

In order to study the phylogenetic relationships among the predicted maize TCPs, we constructed a neighbor-joining tree for the ZmTCP proteins and their orthologs from rice, sorghum and *Arabidopsis*, based on multiple-alignment of the full-length TCP protein sequences (Figure 1). As shown on the phylogenetic tree, the 113 TCPs were classified into two main classes—class I and class II. Class II was further divided into two clades—CYC/TB1 and CIN. The phylogenetic tree based on the sequence alignments of the 46 ZmTCPs was also divided into three clades (Figure S1). The boundaries of these major clades were clearly stated by the phylogenetic locations of several canonical *TCP* genes, such as the class I genes *OsPCF1* and *OsPCF2*, the *CIN*-like class II genes *AtTCP2*, *AtTCP3*, and *AtTCP4*, and the *CYC/tb1*-like class II genes *AtTCP1*, *AtTCP12*, *AtTCP18*, and *ZmTCP2/ZmTB1*. In agreement with previous work, all *Arabidopsis*, rice, and sorghum TCPs fell in the same class or clade, as previously reported in our phylogenetic tree [11,12]. Interestingly, dicot *Arabidopsis* TCP proteins clustered separately from those of the three monocot plants in the same clad, and some proteins from three of the monocot plants displayed pairing. Furthermore, maize and sorghum TCPs were found to share a closer phylogenetic relationship than maize and rice ones, which was consistent with the notion that sorghum is a closer relative of maize than rice. Throughout the phylogenetic tree, there were 49 TCPs in the PCF clade, 34 in CIN, and 30 in CYC/TB1 (Figure 1). Among the 46 *ZmTCPs*, there were 17 *ZmTCPs* in class I and 29 *ZmTCPs* in class II; within class II, 10 in CIN and 19 in CYC/TB1 (Figure S1). Significantly, the number of maize *ZmTCPs* was roughly twice as large as the number in each of rice and sorghum. Specifically, among all 30 CYC/TB1 *TCP* genes, 19 were from maize, 4 from sorghum, and 3 each from rice and *Arabidopsis* (Figure 1). This result suggests that the *ZmTCP* gene family in the allopolyploid maize genome underwent a two-fold duplication. This expansion was biased and occurred mainly in the class II CYC/TB1 clade.

### 2.3. Many TCP Genes are Found in the Syntenic Segments of Rice, Sorghum, and Maize Genomes

**Figure 2:**
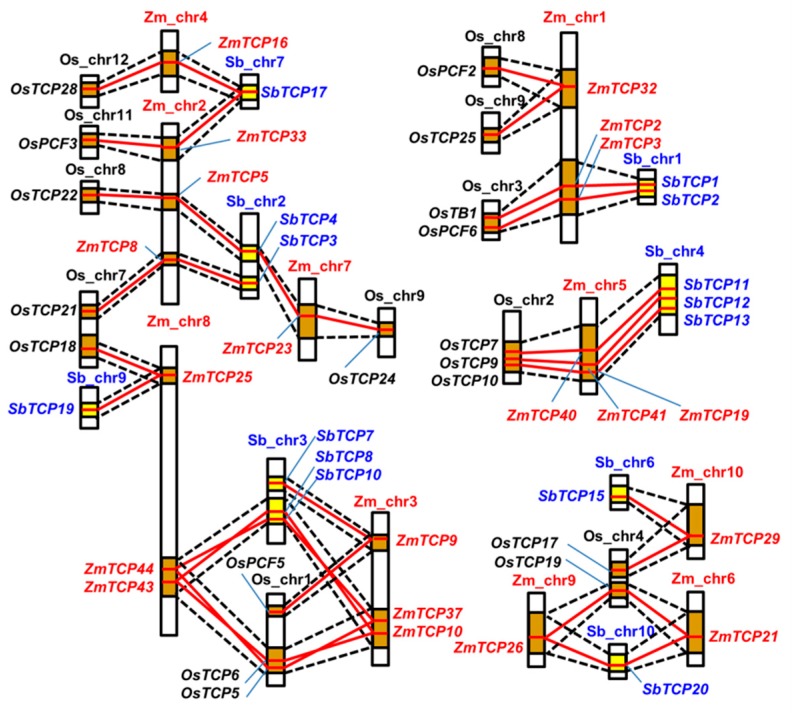
Schematic diagram of syntenic chromosomal segments containing *ZmTCP* genes between the rice, sorghum, and maize genomes. The maize, rice, and sorghum chromosomes are abbreviated Zm, Os, Sb, respectively. Homologous chromosomal regions between the different genomes are linked by black dotted lines and pale blue shaded regions. Each *TCP* orthologous gene pair was connected by a red line. Yellow boxes indicate homologous segments between the maize and the sorghum genomes, while the brown boxes identify the homologous regions in the maize and rice genomes.

As gene synteny is indicative of the homologous gene function, we explored the collinearity of rice, sorghum, and maize *TCP* genes. We collected gene collinearity data from the Plant Genome Duplication Database (PGDD, http://chibba.agtec.uga.edu/duplication, Table S1) using *ZmTCP* genes as anchors, then defined each genomic syntenic block as the chromosomal segment consisting of multiple homologous genes, across species. According to this analysis, about 20 *ZmTCP* genes were found to have syntenic members or collinear genes in rice and sorghum, as shown in Figure 2. Some chromosomal segments containing *ZmTCP* genes, including *ZmTCP2*, *3*, *8*, 9, *19, 25*, *29*, *40*, and *ZmTCP41*, were found to have been evolutionally conserved between maize, rice, and sorghum (Figure 2). This indicated that not only the individual genes but these entire chromosomal segments were evolutionally conserved. Two segments on maize chromosomes 2 and 7, containing a duplicated gene pair *ZmTCP5* and *ZmTCP23,* share synteny with two segments on rice chromosomes 7 and 9, carrying the corresponding genes *OsTCP22* and *OsTCP24*. Two other segments on maize chromosomes 2 and 4, containing another duplicated gene pair *ZmTCP33* and *ZmTCP16,* share synteny with two segments on rice chromosomes 11 and 12, carrying corresponding genes *OsPCF3* and *OsTCP28*. Interestingly, these two paralogous gene pairs, *ZmTCP5*/*ZmTCP23* and *ZmTCP16*/*ZmTCP33*, share the same syntenic blocks on sorghum chromosomes 2 and 7, respectively, indicating that these duplicated gene pairs might have arisen from segmental duplication in maize, after maize and sorghum diverged evolutionarily (Figure 2). One rice chromosomal block, containing *OsTCP19*, shares synteny with two segments on maize chromosomes 6 and 9, with a duplicated gene pair *ZmTCP21* and *ZmTCP26.* However, only one syntenic block could be found in the sorghum genome. Another fragment on rice chromosome 1 containing *OsTCP6* and *OsTCP5* is also duplicated on maize chromosomes 3 and 8, carrying two duplicated gene pairs *ZmTCP10*/*43* and *ZmTCP37/44.* More interestingly, their orthologs in sorghum are also tandemly duplicated on only a single chromosomal segment. Additionally, one maize chromosomal block containing *ZmTCP32* shares synteny with two segments on rice chromosomes 8 and 9, containing its orthologous genes *OsPCF2* and *OsTCP25*; *SbTCP* orthologs could not be found, although the syntenic segment in sorghum was identified. Based on these data, we concluded that most of the *TCP* genes existed before the species diverged, but some *ZmTCP* genes might have originated from later segmental duplication or accompanied the generation of an allotetraploid maize genome.

### 2.4. Expression Profiles of ZmTCP Genes

**Figure 3:**
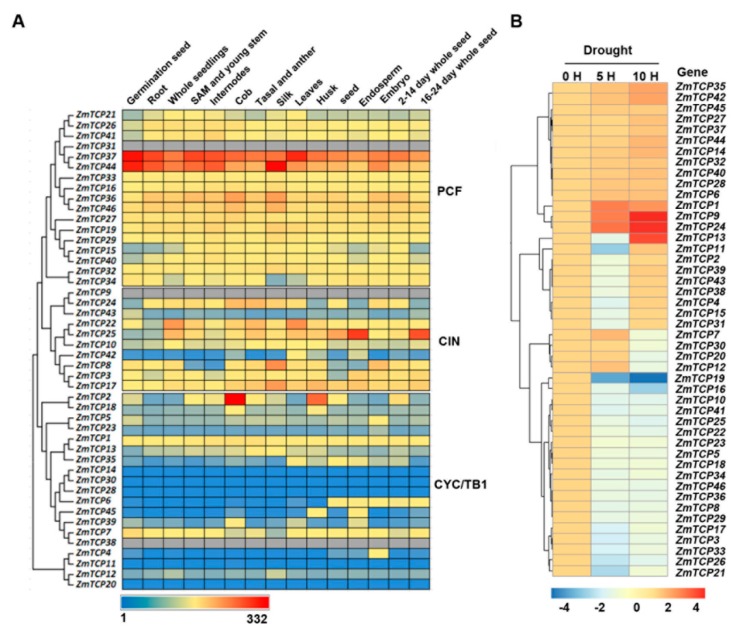
Expression patterns of *ZmTCP* genes in tissues and in response to drought conditions. (**A**) A heat map depicting gene expression levels of 46 *ZmTCPs* in fifteen different tissues from various developmental stages. Normalized gene expression values are shown in different colors that represent the levels of expression indicated by the scale bar. The gray color represents unavailable data. (**B**) Microarray-based expression analysis of *ZmTCP* genes. A heat map was generated based on the fold-change values in the treated samples, when compared with the unstressed control. The color scale for fold-change values is shown at the bottom. The drought-treated leaf samples were collected at two time points, 5 and 10 h, which reflected relative leaf water content (RLWC) of 70% and 60%, respectively.

According to previous studies, *TCP* genes had key roles in different aspects of plant development, as well as in response to stress [8,37]. In order to gain further insights into the roles of the *ZmTCP* genes, we assessed their tissue-specific expression profiles from the available transcriptomic data of maize B73 [43]. An expression heatmap was constructed for the 46 *ZmTCPs* in different tissues, from 15 developmental stages, under non-limiting growth conditions (Figure 3A). Results indicated that the expression patterns of different *ZmTCP* genes varied greatly. Transcripts of *ZmTCP37* and *ZmTCP44* showed relatively high levels of expression, compared to the other *ZmTCPs* examined. It is noteworthy that *ZmTCP2/TB1* and *ZmTCP18,* a homolog of *OsTB1*, were expressed relatively high in cobs and husk leaves, while *ZmTCP25* was expressed relatively high in endosperm and mature seeds. Additionally, in the B73 variety grown under non-limiting conditions, all *ZmTCP* genes in class I (PCF clade) were expressed relatively high in different tissues.

Next, we investigated the expression profiles of all maize *TCP* genes, in response to drought stress, using microarray analysis. Based on their expression patterns, the maize *TCP* genes could mainly be classified into four groups (Figure 3B). The expression levels of 14 *ZmTCP* genes were continuously up-regulated (fold-change > 1) in response to both drought stress conditions (5 h and 10 h of drought treatments). Among these genes, *ZmTCP1*, *ZmTCP9*, and *ZmTCP24* showed two-fold changes (or more) in one or two drought stress conditions. In contrast, 19 *TCP* genes were continuously down-regulated (fold-change < −1) under both drought stress conditions, of which *ZmTCP19* showed about four fold change in one or two drought stress conditions. Expression of the remaining 13 genes, were either suppressed or induced under one of the drought conditions. The results clearly showed the functional divergence of *ZmTCP* genes in response to drought stress, in maize seedlings.

### 2.5. Association Analysis of Natural Variations in ZmTCP Genes Identified Two ZmTCP Genes Associated with Drought Tolerance in Maize

In order to further investigate whether the natural variations in any of the ZmTCP TFs are associated with the different drought tolerance levels of maize varieties, we conducted an association analysis for these genes. The drought tolerance level of each inbred line was investigated by evaluating its survival rate under severe drought stress, at the seedling stage. To assess potential associations between survival rates and *ZmTCPs*, we utilized previously reported methods and data [44,45] and previously identified single nucleotide polymorphism (SNP) markers, to characterize the presence of genetic polymorphisms in each of these 46 *ZmTCP* genes. Among the 46 identified *ZmTCP* genes, 26 were found to be polymorphic with an average of 12 SNPs (Table 2), while the polymorphic information of the other 16 genes was currently absent (Minor Allele Frequency, MAF ≥ 0.05).

**Figure 4:**
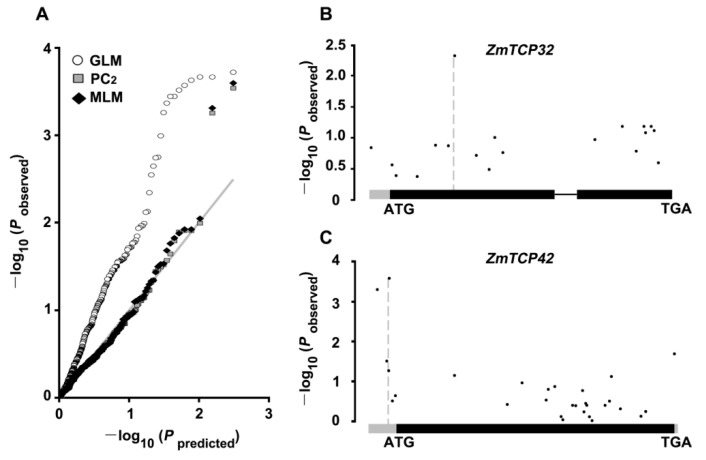
Association analysis of genetic variations in *ZmTCP32 and ZmTCP42* with maize drought tolerance. (**A**) Quantile–quantile plots of the estimated −log10(*p*) from the *ZmTCP* gene family-based association analysis, using three methods. The gray line is the expected line under the null distribution. The white square represents the observed *p* values using general linear model (GLM); the gray square represents the GLM model with the first two principal components (PC_2_); the black diamond represents the observed *p* values using the mixed linear model (MLM) model incorporating both PC_2_ and a Kinship matrix. (**B**,**C**) Schematic diagrams of *ZmTCP32* (**B**) and *ZmTCP42* (**C**), including the UTR (gray), intron (thin black line), and protein coding regions (thick black line), are presented in the x-axis. The *p* value is shown on a −log10 scale.

*ZmTCP37* was found to be the most polymorphic, with 34 SNPs in this natural diversity panel. Subsequently, three statistical models were applied to identify significant genotypic and phenotypic associations. Specifically, a general linear model (GLM) with the first two principal components (PC_2_) and a mixed linear model (MLM) were used to find associations (Figure 4A). The GLM method was applied to perform single-marker analysis. Then PC_2_, via the first two principal components of the SNP data, was applied to correct for spurious associations caused by the population structure. The MLM method, incorporating both PC_2_ and a Kinship matrix (to correct for the effect of cryptic relatedness), was considered to be effective for controlling false positives in the association analysis (Figure 4A) [46,47]. The candidate gene association analysis detected significant associations between the genetic variations of *ZmTCP32* and *ZmTCP42* and drought tolerance, under different models, with a *p*-value ≤ 0.01 (Table 2; Figure 4B,C). However, under the standard mixed linear model (MLM), the only two significantly associated SNPs contributing to the phenotype of drought tolerance were both located at the 5′ UTR region of *ZmTCP42*, which suggested that this candidate gene was significantly associated with drought tolerance (*p*-value ≤ 0.001, −log_10_*p*  =  3.77) (Figure 4C). We further analyzed the survival rates of maize inbred lines carrying *ZmTCP42* drought-tolerant or drought-sensitive alleles. It was found that *ZmTCP42^AA^* was a favorable drought tolerance allele (Figure S2). The two-fold induction of *ZmTCP42* expression by dehydration (Figure 3B) suggested that the RNA level of *ZmTCP42* was likely more correlated with drought tolerance than other potential variations among different maize varieties.

### 2.6. ZmTCP32 and ZmTCP42 Are Both Induced by ABA Treatment and Drought Stress

**Figure 5:**
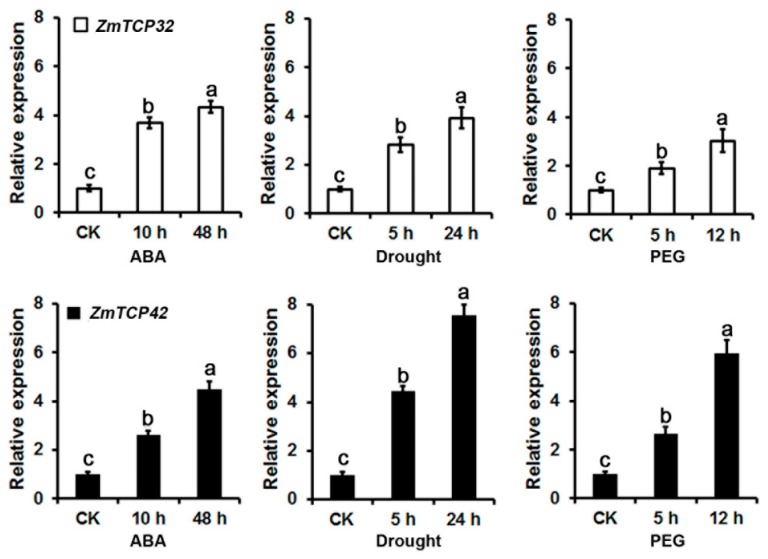
The RNA levels of *ZmTCP32* and *ZmTCP42* in maize B73 leaves measured by RT-qPCR, after treatment with 100  μM ABA, drought stress, or 10% PEG treatment. The drought stress was performed as previously described [45], on hydroponically cultured seedlings, for 5 and 24 h, and their relative leaf water contents (RLWC) were determined to be approximately 70% and 58%, at the corresponding time points, respectively. *ZmUbi-2* transcript levels were used as an internal control for data normalization; the gene-specific primers are listed in Table S2. Each data point represents the mean ± SD (*n* = 3) of three biological replicates. Significant differences were calculated by one-way ANOVA with Duncan’s multiple test (SAS Institute, Inc., Cary, NC, USA). Different letters indicate a significant statistical difference between sample means, at *p*  =  0.05, while means with the same letters were not significantly different.

To confirm whether *ZmTCP32* and *ZmTCP42* RNA levels are truly associated with drought tolerance, we used RT-qPCR to directly analyze the RNA expression of the *ZmTCP32* and *ZmTCP42* genes, in response to ABA treatment and drought stress. As illustrated in Figure 5, in the B73 genotype, *ZmTCP32* and *ZmTCP42* are both significantly induced by ABA by roughly four-fold, relative to the controls at 48 h after the ABA treatment. More importantly, *ZmTCP42* was highly induced by drought stress (+7.6-fold at 24 h) and by the PEG6000 treatment (+5.9-fold at 24 h); *ZmTCP42* was significantly more responsive to dehydration stress and PEG treatment than *ZmTCP32*, even though *ZmTCP32* was also responsive to drought stress (+3.9-fold at 24 h) and PEG treatment (+3.0-fold at 24 h) (Figure 5).

### 2.7. Overexpression of ZmTCP42 Enhances Drought Resistance in Transgenic Arabidopsis

**Figure 6:**
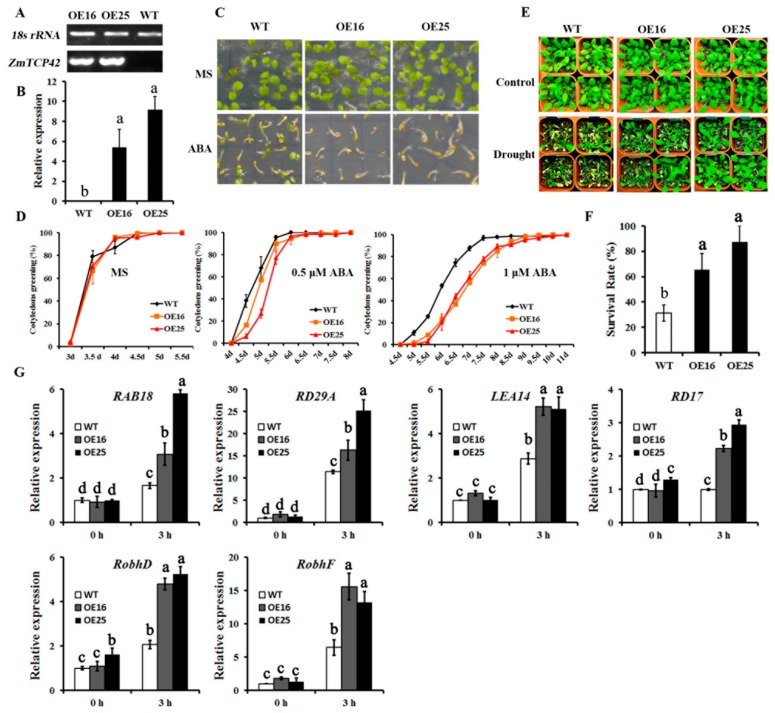
Analysis of wild-type (WT) and *ZmTCP42-*overexpressing transgenic *Arabidopsis* plants. (**A**) RT-PCR analysis of *ZmTCP42* transcript levels in *ZmTCP42* transgenic lines. (**B**) RT-qPCR analysis of *ZmTCP42* transcript levels in *ZmTCP42* transgenic lines. (**C**) Seed germination of the *ZmTCP42* transgenic lines and in the wild-type, in response to abscisic acid (ABA). Germination rates with 1  μM ABA for 5 days, defined by cotyledon greening, in wild-type *ZmTCP42-OE16* and *ZmTCP42-OE25*, compared to the MS medium alone. (**D**) Statistical analysis of a green seedling’s rate of *ZmTCP42* transgenic lines and wild-type grown on Murashige and Skoog (MS) medium with 0, 0.5  μM, and 1  μM ABA. (**E**) Drought tolerance of the *ZmTCP42* transgenic Arabidopsis plants. Photographs were taken before and after the drought treatment, followed by 3 days of rewatering. (**F**) Statistical analysis of survival rates after the drought-stress treatment. The average survival rates and standard errors were calculated from three independent experiments. (**G**) RNA levels of stress-responsive genes in the *ZmTCP42* transgenic lines and in the wild-type, in response to drought stress. Total RNA was obtained from 3 weeks-old seedlings treated by 3 h of dehydration stress and was analyzed by RT-qPCR, using the gene-specific primers listed in Table S2. The mean value and standard error were calculated from three replicates after normalization to *ACTIN2*. The RNA levels in the wild-type grown under non-stress conditions was taken as 1.0. Significant differences were calculated by one-way ANOVA with Duncan’s multiple test (SAS Institute, Inc., Cary, NC, USA). Different letters indicate a significant statistical difference between the sample means at *p*  =  0.05, while means with the same letters were not significantly different.

Given that the polymorphism in the 5′-UTR of *ZmTCP42* suggests a potential role in drought resistance on maize seedlings, we selected *ZmTCP42* to directly test for its function. To validate *ZmTCP42* function in response to drought stress, we generated transgenic Arabidopsis plants that overexpress the *ZmTCP42* gene, by using the enhanced cauliflower mosaic virus 35S promoter. After screening for the *ZmTCP42* expression levels, we selected two independent transgenic lines, *ZmTCP42-OE16* and *ZmTCP42-OE25*, with enhanced RNA levels, for further experiments (Figure 6A,B). We investigated the ABA sensitivity of *ZmTCP42* transgenic lines, in response to exogenous ABA, during seed germination. In the absence of exogenous ABA, all lines germinated completely, as did the wild-type seeds; on the contrary, in the presence of exogenous ABA, the expanding, greening cotyledons were significantly affected in the *ZmTCP42*-OE lines; the number of seedlings with green cotyledons in *ZmTCP42-OE16* and *ZmTCP42-OE25* were significantly lower than those of the wild-type at 1 µM ABA (Figure 6C,D), suggesting that the *ZmTCP42* overexpression lines are more sensitive to ABA. Furthermore, we analyzed the tolerance levels of *ZmTCP42*-OE lines to drought stress. When the 28-day-old transgenic seedlings were subjected to the drought test, only ~30% of the wide-type plants were able to recover from the stress, whereas ~65 of *ZmTCP42-OE16* and ~87% of *ZmTCP42-OE25* transgenic plants survived (Figure 6E,F). Similar results were obtained in repeated experiments, indicating that overexpression of *ZmTCP42* enhanced drought tolerance in Arabidopsis.

We further assessed the expression levels of ABA- inducible or drought-inducible genes by using real-time RT-qPCR to analyze their responses in *Arabidopsis*. Upon dehydration stress, the RNA levels of *RAB18*, *RD29A*, *LEA14*, and *RD17*, which are well-known drought-responsive, positive regulator genes of drought tolerance, were up-regulated, relative to wild-type *Arabidopsis* plants (Figure 6G). Similar changes were observed for the RNA levels of the *RbohD* and *RbohF* genes, encoding two NADPH oxidases, which were directly responsible for the reactive oxygen species (ROS) production in leaves, in response to stress (Figure 6G). These results clearly showed that *ZmTCP42* overexpression improved the inducibility of *Arabidopsis* drought-tolerance-associated genes upon drought stress, leading to an enhanced drought tolerance.

## 3. Discussion

To date, the TCP family members have been described in various species; for instance, 24 *TCP* genes in *Arabidopsis*, 23 in rice, 20 in sorghum, and 66 in wheat (*Triticum aestivum* L.) [11,12,16,17]. Chai et al. (2017) reported 29 *ZmTCP* genes in maize, including their chromosomal location, structure and domain conservation analysis, and gene duplication analysis. They also analyzed the phylogenetic inference and expression profiling of these 29 *ZmTCP* genes, and then speculated that the *ZmTCP* genes influence stem and ear growth. In our study, we retrieved a total of 46 *ZmTCP* genes, by searching against the updated maize genome B73_RefGen_v3, including the 29 *ZmTCPs* previously reported by Chai et al. and the 17 newly identified *ZmTCPs* containing a canonical TCP domain. Subsequently, we further systematically determined their phylogenetic relationship, synteny with rice, sorghum, and Arabidopsis *TCPs*, pattern of drought-responsiveness, association analysis of their natural variations with drought tolerance, and functional analysis of *ZmTCP42* in drought tolerance. Collectively, our data demonstrated that a few *ZmTCP* family proteins are likely involved in plant drought tolerance; in particular, ZmTCP42 functions as an important positive regulator for drought tolerance.

Recently, genome-wide identification revealed that segmental duplication might be the main contributor to the expansion of the *TCP* gene family in some monocots, including rice, sorghum and wheat, as well as in some dicot species, such as cotton and soybean [14,17,48,49,50,51,52]. The report showed that there were about 2.6–2.8-fold duplication of the *TCP* gene family in allotetraploid upland cotton genome (*G. hirsutum*), compared to *Arabidopsis* [48]. Similarly, we found that the duplication ratio of the *ZmTCP* family was about 2-fold in the allopolyploid maize genome, compared with *Arabidopsis*, rice, and sorghum. The change in the ratio of gene numbers suggests that the *ZmTCP* family has undergone lineage-specific expansion and functional divergence during the course of evolution. Furthermore, as shown in the phylogenetic analysis, monocot ZmTCP proteins clustered independently from those in *Arabidopsis*, suggesting a potential functional divegence between the dicot and monocot TCPs. We also found that *ZmTCP* gene expansion was not uniform, and occurred mainly in the class II CYC/TB1 clade. However, the biological significance of the *ZmTCP* gene duplication in the maize genome remains to be determined. Our collinearity analysis on *TCP* genes within several monocot plants showed that 20 *ZmTCP* genes had syntenic members or collinear genes in rice and sorghum, and that the chromosomal segments containing these genes were also duplicated (Figure 2), supporting the concept that the maize genome might have arisen from an ancestral allotetraploid, half of which share a common ancestor with sorghum, which in turn probably represents a lineage split from rice [53,54]. Taken together, our results might provide some useful clues for future studies on a homologous gene function.

A growing body of research suggest that TCP TFs play important roles in plant development, as well as in response to abiotic stress [8,19,33,34]. However, the role of *TCP* genes in plant response to drought stress in maize was still obscure, without answers to which, the *ZmTCP* genes were directly associated with the levels of drought tolerance in maize. We were, therefore, prompted to perform this study to address this question. The answer would not only help facilitate the genetic improvement of drought tolerance but would also increase our knowledge of the biological function of this gene family. Generally, *ZmTCP* genes exhibit great differential expression in response to drought stress in maize seedlings, not only among subgroups but members within the same subgroup, suggesting that these *ZmTCP* genes might function very diversely. Our current results showed that 14 *ZmTCP* genes were significantly up-regulated and 19 genes were clearly down-regulated, indicating that these genes might function as key mediators of drought stress responses in maize (Figure 3). Notably, three genes, *ZmTCP1*, *ZmTCP9*, and *ZmTCP24*, belonging to the class II CYC/TB1 clade, showed similar inducible expression patterns (Figure 3), implying that they might play a redundant role in regulating drought stress response in maize. Moreover, the orthologs of *ZmTCP9* and *ZmTCP24* in rice and *Arabidopsis* are *OsPCF5* and *AtTCP3*/*AtTCP4*, respectively, which are regulated by the conserved miR319. Previous reports have shown that knockdown of miR319-dependent TCPs (by constitutively overexpressing miR319) increased drought and salinity tolerance [34,55]. The rice homolog of *ZmTCP1* and *ZmTCP13* was named *OsTB1*, which plays an important role in stress response, especially in regulation of cold tolerance [55,56]. Additionally, *ZmTCP37* and *ZmTCP44*, orthologs of *AtTCP20*, also showed a slightly inducible expression pattern under drought stress (Figure 3). In *Arabidopsis*, *AtTCP20* represses the transcription of *LIPOXYGENASE2* (*AtLOX2*) gene, which is involved in jasmonic acid synthesis and promotes leaf senescence [57].

To explore the association of *TCPs* with stress tolerance in plants, we have investigated homologous *ZmTCP* genes in a small number of inbred lines and proposed predictive conclusions. Among the 46 *ZmTCP* genes analyzed, the genetic polymorphisms of *ZmTCP32* and *ZmTCP42* were significantly associated with the phenotypic variations of drought tolerance (*p*-value ≤ 0.01, PC_2_) (Table 2). While *ZmTCP42* was the most significantly (*p*-value ≤ 0.001, MLM) associated with drought tolerance in this natural variation panel, the natural variation in the *ZmTCP42* promoter might contribute to maize drought tolerance (Figure 4). In the B73 genotype of maize, *ZmTCP32* was constitutively highly expressed in various tissues (Figure 3A). In comparison with *ZmTCP32*, *ZmTCP42* RNA was detected at a low level in various tissues, except in leaves and endosperm, and showed a relatively high level at 10 h, upon drought stress in a micrroarry analysis (Figure 3B). Both *ZmTCP32* and *ZmTCP42* were significantly induced by ABA, dehydration and PEG treatment; more notably, *ZmTCP42* was induced higher than *ZmTCP32* in response to dehydration and PEG treatment. These results suggested that *ZmTCP42* was involved in plant drought response. Transgenic *Arabidopsis* overexpressing *ZmTCP42* exhibited a higher ABA sensitivity, improved drought stress tolerance, and enhanced the induction of Arabidopsis ABA- or drought-inducible genes, which strongly suggests that ZmTCP42 might regulate these ABA/drought-response genes. In summary, we have identified ZmTCP42 as an important positive regulator of drought tolerance, through analyses of gene expression and natural variations.

## 4. Materials and Methods

### 4.1. Plant Growth Conditions and Stress Treatments

Maize (Inbred line B73) growth conditions and stress treatments were performed according to Wang et al. [45]. The hydroponically cultured 3-leaf stage seedlings were used. For drought treatments, the seedlings were placed on a clean bench and subjected to dehydration (at 28 °C, with a relative humidity of 40–60%). Samples were exposed for 0, 5 h, and 24 h, at which the relative leaf water contents were measured (RLWC) to be 98%, 70%, and 60%, respectively, in the drought-treated leaf samples. ABA and PEG treatments were applied by immersing the seedlings in 100 μM ABA or 10% PEG 6000. All leaf samples from a minimum of 3 seedlings were immediately frozen in liquid nitrogen, then stored at −80 °C, prior to RNA isolation. The *Arabidopsis thaliana* ecotype Col-0 was used in this study. Seeds were grown on the Murashige and Skoog (MS) medium with an addition of 3% sucrose and 0.6% agar (pH 5.8). Drought treatment was applied to the 3-week-old seedlings by removing them from the MS medium plates and desiccating them on Whatman 3MM paper, on a clean bench, for 0 h and 3 h, respectively.

### 4.2. Reverse Transcription PCR and RT-qPCR Analysis

Total RNA was isolated from the collected leaf samples using the TRIZOL reagent (Biotopped, China) according to the manufacturer’s instructions. One microgram of the total RNA from each sample was used in reverse transcription. RT-qPCR analyses were performed in the optical 48-well plates, using the ABI7300 Thermo-cycler (Applied Biosystems, USA). Reactions were carried out in a 10 μL volume containing 1 μL diluted cDNA, 200 nM gene-specific primers, and 5 μL SYBR Premix Ex Taq II (Takara, China) with the following conditions—10 min at 95 °C, 40 cycles of 15 s at 95 °C, and 30 s at 60 °C. Each experiment was performed using at least three independent biological replicates. *ZmTCP32* and *ZmTCP42* RNA levels in response to different stresses were calculated according to a standard curve calibration, based on amplification of a dilution series of *ZmTCP32* and *ZmTCP42* plasmid, respectively, according to the manufacturer’s protocol. *ZmUbi-2* (UniProtKB/TrEMBL; ACC: Q42415) was used as the internal control. The expression levels of different stress-responsive genes were compared, based on the delta Ct method with normalization to *ACTIN2*.

### 4.3. Generation of Transgenic Plants

The coding region of the *ZmTCP42* cDNA of the maize B73 inbred line was inserted into the pGreenII vector [58]. The constructed plasmid carrying the desired gene was transformed into *Agrobacterium tumefaciens* GV3101 + pSoup and transformed into ecotype Col-0, as described previously [58]. Using the kanamycin-based selection, several independent T_2_ transgenic lines were obtained, and expression of the *ZmTCP42* transgene was confirmed in these lines by RT-PCR and RT-qPCR. Two independent overexpression lines *ZmTCP42-OE16* and *ZmTCP42-OE25* (with a single inserted copy) were selected, based on the level of transgene expression, and the T3 homozygous seeds were subjected to further analyses.

### 4.4. Seed Germination and Drought Phenotype Analysis

To study the effect of ABA on germination and cotyledon greening, the seeds were planted on MS medium plates, with an addition of plus 1% sucrose and different concentrations of ABA (Sigma-Aldrich) in a growth chamber, at 22 °C under a 16-h-light/8-h-dark photo-period, with a 60% relative humidity, after 2 days of vernalization in darkness at 4 °C. For the drought stress tolerance test, plants were grown for 28 days under normal conditions and subjected to water stress by withholding watering for 14 days [58]. Four plants were planted in each small cup with 100 g soil (1:1 of black soil/vermiculite) and grown under the condition of 16-h-light/8-h-dark. Four independent experiments were performed and at least 16 plants each were observed, for the *ZmTCP42-OE16* and *ZmTCP42-OE25* lines and the WT, in each experiment. The plants were rewatered when significant differences in wilting were observed. Three days after rewatering, the surviving plants were counted.

### 4.5. Identification of the TCP Proteins in the Maize Genome

To identify TCP proteins in the B73 maize genome, some putative TCP protein sequences from maize (genome assembly: B73_RefGen_v3) were first retrieved from the Plant Transcription Factor Database 3.0 (Jin et al. 2014). Conserved TCP DNA-binding domain (PF03634) from the Pfam database [59] was used to search and retrieve sequences from the Phytozome database v10.0 (available online: http://www.phytozome.net/eucalyptus.php). In addition, BLASTP searches were also performed against the maize genome, to identify any additional TCP members using *Arabidopsis* and rice TCP proteins sequences (*E-value* ≤ 0.00001). The overlapped genes were removed. The presence of a TCP domain in all family proteins was evaluated using the CDD database searches (https://www.ncbi.nlm.nih.gov/Structure/cdd). Arabidopsis TCP proteins were downloaded from TAIR 10 (available online: http://www.arabidopsis.org), which contained 24 members. TCP proteins in rice (Genome assembly: Rice Genome Annotation Project Database release 7) and sorghum (Genome assembly: V3) were both downloaded from the Plant Transcription Factor Database. The ExPASy program (http://www.expasy.org/tools/) was used to predict the molecular weight (kDa) and the isoelectric point (PI) of each protein.

### 4.6. Gene Structure and Phylogenetic Analysis

Genomic sequences of maize TCP genes were downloaded from Phytozome V10, and the untranslated regions were removed. To show the exon/intron organization for individual TCP genes, coding sequences were aligned to genomic sequences and schematics generated using GSDS 2.0 (Gene Structure Display Server) (available online: http://gsds.cbi.pku.edu.cn) [60]. To determine the phylogenetic relationships of the TCP proteins, full-length amino acid sequences of TCPs identified in maize, rice, *Arabidopsis*, and sorghum were aligned by the ClustalW program [61]. The Phylogenetic tree was constructed by the neighbor-joining method, with 1000 bootstrap replicates.

### 4.7. Association Analysis

Association analysis for *ZmTCPs* was performed by using a maize association mapping population containing 367 inbred lines and the corresponding drought tolerance phenotype data from a previous study [45]. Among the 556,945 high-quality single nucleotide polymorphism (SNP) data, with minor allele frequency (MAF) ≥ 0.05, 317 SNPs were found in the genic region of the 46 *ZmTCPs*. The general linear model (GLM) model, the GLM model with the first 2 principal components (PC_2_), and the mixed linear model (MLM) model were chosen to detect the SNPs significantly associated with drought tolerance, by using the TASSEL5.0 program [62].

### 4.8. Tissue Expression Profile and Microarray Analysis

Expression patterns of 46 *ZmTCPs* in different maize tissues were analyzed using the genome-wide gene expression atlas of the inbred B73 line of maize that was reported previously [43]. Expression data for the 15 tissues were combined from 60 growth stages. Normalized expression values of each gene in different tissues were averaged. The gene expression level was presented as a log value. To analyze the gene expression patterns of *ZmTCPs* in maize during drought stress, the microarray data and analysis methods were employed as described previously [63].

## Figures and Tables

**Table 1:** Detailed information for 46 *ZmTCP* genes in the *Zea mays* L. genome.

Gene Name	Locus ID	Chr	CDS Length (bp)	Protein Length (aa)	Number of Exons	MW	pI	Class
*ZmTCP1*	GRMZM2G166687	1	723	240	1	26.11	5.15	CYC/TB1
*ZmTCP2*	AC233950.1_FG002	1	1131	376	1	39.86	7.8	CYC/TB1
*ZmTCP3*	GRMZM2G115516	1	1161	386	2	39.36	9.33	CIN
*ZmTCP4*	AC234521.1_FG006	2	768	255	2	27.71	10.62	CYC/TB1
*ZmTCP5*	GRMZM2G110242	2	831	276	1	29.9	6.26	CYC/TB1
*ZmTCP6*	GRMZM2G088440	2	549	182	5	21.14	8.65	CYC/TB1
*ZmTCP7*	GRMZM2G414114	2	1104	367	6	42.29	8.44	CYC/TB1
*ZmTCP8*	GRMZM2G020805	2	1323	440	2	46.22	9.46	CIN
*ZmTCP9*	AC205574.3_FG006	3	1698	565	2	58.83	7.1	CIN
*ZmTCP10*	GRMZM2G166946	3	852	283	2	30.48	8.98	CIN
*ZmTCP11*	GRMZM2G055024	4	1263	420	3	45.5	10.82	CYC/TB1
*ZmTCP12*	GRMZM2G062711	4	564	187	1	21.58	12.05	CIN
*ZmTCP13*	GRMZM2G060319	4	825	274	1	29.35	5.96	CYC/TB1
*ZmTCP14*	GRMZM2G135461	4	402	132	3	15.09	7.32	CYC/TB1
*ZmTCP15*	GRMZM2G078077	4	657	218	1	22.97	10.02	PCF
*ZmTCP16*	GRMZM2G003944	4	1164	387	1	40.76	9.33	PCF
*ZmTCP17*	GRMZM2G089361	5	1146	381	1	39.05	9.58	CIN
*ZmTCP18*	AC190734.2_FG003	5	1080	359	1	38.89	7.01	CYC/TB1
*ZmTCP19*	GRMZM2G445944	5	624	207	1	21.12	10.26	PCF
*ZmTCP20*	GRMZM2G031905	6	435	144	3	16.01	9.89	CIN
*ZmTCP21*	GRMZM2G142751	6	1140	379	1	39.09	8.02	PCF
*ZmTCP22*	GRMZM2G120151	6	897	298	2	30.9	8.07	CIN
*ZmTCP23*	GRMZM2G064628	7	843	280	1	30.23	6.33	CYC/TB1
*ZmTCP24*	GRMZM2G015037	8	1974	657	4	69.55	6.61	CIN
*ZmTCP25*	GRMZM2G035944	8	828	275	2	29.59	9.02	CIN
*ZmTCP26*	GRMZM2G113888	9	1209	402	1	40.66	8.81	PCF
*ZmTCP27*	GRMZM2G096610	10	531	176	1	18.33	7.43	PCF
*ZmTCP28*	GRMZM2G458087	10	495	164	3	18.83	8.37	CYC/TB1
*ZmTCP29*	GRMZM2G465091	10	624	207	1	21.01	10.01	PCF
*ZmTCP30*	GRMZM2G454571	1	399	132	3	15.11	7.27	CYC/TB1
*ZmTCP31*	AC213524.3_FG003	1	831	276	2	29.56	9.83	PCF
*ZmTCP32*	GRMZM2G107031	1	972	323	2	34.02	5.98	PCF
*ZmTCP33*	GRMZM2G416524	2	894	297	3	32.37	10.43	PCF
*ZmTCP34*	AC199782.5_FG003	2	1032	343	1	35.43	5.13	PCF
*ZmTCP35*	GRMZM5G824514	3	297	98	1	11.76	12.23	CYC/TB1
*ZmTCP36*	GRMZM2G089638	3	1251	416	1	42.91	5.79	PCF
*ZmTCP37*	GRMZM2G092214	3	975	324	2	34.35	6.7	PCF
*ZmTCP38*	GRMZM2G359599	4	522	173	4	19.58	8.6	CYC/TB1
*ZmTCP39*	GRMZM2G170232	4	396	131	4	14.38	8.87	CYC/TB1
*ZmTCP40*	GRMZM2G178603	5	663	220	1	22.76	10.02	PCF
*ZmTCP41*	GRMZM2G077755	5	1200	400	1	40.49	9.2	PCF
*ZmTCP42*	GRMZM2G180568	7	972	323	1	33.71	8.11	CIN
*ZmTCP43*	GRMZM2G148022	8	2337	778	15	84.78	7.35	CIN
*ZmTCP44*	GRMZM2G034638	8	948	315	2	33.08	6.57	PCF
*ZmTCP45*	GRMZM2G424261	10	489	162	3	17.27	5.91	CIN
*ZmTCP46*	GRMZM2G093895	10	1218	405	1	41.09	5.95	PCF

**Table 2:** Association analysis of the natural variation in *ZmTCP* genes with respect to drought tolerance at the seedling stage in the maize diversity panel.

Locus ID	Gene Name	Polymorphic Name	GLM	PCA	PCA + K	
			*p* ≤ 0.01	*p* ≤ 0.01	*p* ≤ 0.01	*p* ≤ 0.001
GRMZM2G166687	*ZmTCP1*	-	-	-	-	-
AC233950.1_FG002	*ZmTCP2*	3	2	0	0	0
GRMZM2G115516	*ZmTCP3*	33	0	0	0	0
AC234521.1_FG006	*ZmTCP4*	-	-	-	-	-
GRMZM2G110242	*ZmTCP5*	1	0	0	0	0
GRMZM2G088440	*ZmTCP6*	-	-	-	-	-
GRMZM2G414114	*ZmTCP7*	-	-	-	-	-
GRMZM2G020805	*ZmTCP8*	21	0	0	0	0
AC205574.3_FG006	*ZmTCP9*	19	0	0	0	0
GRMZM2G166946	*ZmTCP10*	1	0	0	0	0
GRMZM2G055024	*ZmTCP11*	-	-	-	-	-
GRMZM2G062711	*ZmTCP12*	-	-	-	-	-
GRMZM2G060319	*ZmTCP13*	1	0	0	0	0
GRMZM2G135461	*ZmTCP14*	-	-	-	-	-
GRMZM2G078077	*ZmTCP15*	5	0	0	0	0
GRMZM2G003944	*ZmTCP16*	-	-	-	-	-
GRMZM2G089361	*ZmTCP17*	28	1	0	0	0
AC190734.2_FG003	*ZmTCP18*	-	-	-	-	-
GRMZM2G445944	*ZmTCP19*	7	0	0	0	0
GRMZM2G031905	*ZmTCP20*	-	-	-	-	-
GRMZM2G142751	*ZmTCP21*	2	0	0	0	0
GRMZM2G120151	*ZmTCP22*	2	0	0	0	0
GRMZM2G064628	*ZmTCP23*	-	-	-	-	-
GRMZM2G015037	*ZmTCP24*	2	0	0	0	0
GRMZM2G035944	*ZmTCP25*	27	1	0	0	0
GRMZM2G113888	*ZmTCP26*	4	0	0	0	0
GRMZM2G096610	*ZmTCP27*	12	0	0	0	0
GRMZM2G458087	*ZmTCP28*	-	-	-	-	-
GRMZM2G465091	*ZmTCP29*	1	0	0	0	0
GRMZM2G454571	*ZmTCP30*	-	-	-	-	-
AC213524.3_FG003	*ZmTCP31*	-	-	-	-	-
GRMZM2G107031	*ZmTCP32*	19	0	1	1	0
GRMZM2G416524	*ZmTCP33*	-	-	-	-	-
AC199782.5_FG003	*ZmTCP34*	12	0	0	0	0
GRMZM5G824514	*ZmTCP35*	-	-	-	-	-
GRMZM2G089638	*ZmTCP36*	2	0	0	0	0
GRMZM2G092214	*ZmTCP37*	34	3	0	0	0
GRMZM2G359599	*ZmTCP38*	-	-	-	-	-
GRMZM2G170232	*ZmTCP39*	-	-	-	-	-
GRMZM2G178603	*ZmTCP40*	2	1	0	0	0
GRMZM2G077755	*ZmTCP41*	-	-	-	-	-
GRMZM2G180568	*ZmTCP42*	29	2	2	2	2
GRMZM2G148022	*ZmTCP43*	1	0	0	0	0
GRMZM2G034638	*ZmTCP44*	22	6	0	0	0
GRMZM2G424261	*ZmTCP45*	-	-	-	-	-
GRMZM2G093895	*ZmTCP46*	27	2	0	0	0

## Supplementary Materials

Supplementary materials can be found at https://www.mdpi.com/1422-0067/20/11/2762/s1.

## Abbreviations

TCP	the teosinte branched1/cycloidea/proliferating
TF	transcription factor
CYC/TB1	cycloidea/teosinte branched 1
PCF	proliferating cell nuclear antigen factor
CIN	CINCINNATA of Antirrhinum
RT-qPCR	reverse transcription quantitative polymerase chain reaction
GLM	general linear model
SNP	single nucleotide polymorphism
MLM	mixed linear model
ABA	abscisic acid
PEG	polyethylene glycol
